# Bone marrow contribution to synovial hyperplasia following joint surface injury

**DOI:** 10.1186/s13075-016-1060-8

**Published:** 2016-07-13

**Authors:** Ana Sergijenko, Anke J. Roelofs, Anna H. K. Riemen, Cosimo De Bari

**Affiliations:** Arthritis and Regenerative Medicine Laboratory, Institute of Medical Sciences, University of Aberdeen, Foresterhill, Aberdeen, AB25 2ZD UK

**Keywords:** Mesenchymal stem cells, Stromal cells, Synovium, Bone marrow transplantation, Joint injury

## Abstract

**Background:**

Joint surface injury, a known risk factor for osteoarthritis, triggers synovial hyperplasia, which involves proliferation of mesenchymal stromal/stem cells (MSCs). Whether these proliferative MSCs are resident synovial cells or move into the tissue from elsewhere is not known. The aim of this study was to determine the contribution of bone marrow-derived cells to synovial hyperplasia following joint surface injury.

**Methods:**

Lethally irradiated mice were transplanted with green fluorescent protein (GFP)-labelled bone marrow, and MSC chimerism was determined by the colony-forming unit fibroblast (CFU-F) assay and phenotypic analysis. To label host slow-cycling cells prior to bone marrow transplant, mice received iododeoxyuridine for 3 weeks. Mice then were subjected to GFP^+^ bone marrow transplant, underwent joint surface injury and received chlorodeoxyuridine (CldU) for 7 days to label cells proliferating after injury. GFP- and nucleoside-labelled cells in normal and injured knee joint synovium were quantified in situ by immunofluorescence staining of paraffin-embedded tissue sections. The phenotype of GFP-labelled cells was determined by co-staining for the haematopoietic marker CD16/CD32 and the MSC/fibroblast marker platelet-derived growth factor receptor α (Pdgfrα).

**Results:**

CFU-F assay and phenotypic analysis demonstrated successful bone marrow mesenchymal lineage chimerism in mice that underwent transplants. Bone marrow reconstitution preceded the detection of GFP-labelled cells in synovium. The percentage of GFP^+^ cells in synovium increased significantly in response to injury, while the proportion of GFP^+^ cells that were labelled with the proliferation marker CldU did not increase, suggesting that the expansion of the GFP^+^ cell population in synovium was due mainly to bone marrow cell infiltration. In contrast, proliferation of host slow-cycling cells was significantly increased in the hyperplastic synovium. In both control and injured knee joints, the majority of marrow-derived GFP^+^ cells in the synovium were haematopoietic (CD16/32^+^), while a minority of cells expressed the pan-fibroblast/MSC marker Pdgfrα.

**Conclusions:**

Our findings indicate that synovial hyperplasia following joint surface injury involves proliferation of resident slow-cycling cells, with a contribution from infiltrating bone marrow-derived cells. Understanding the process of synovial hyperplasia may reveal ways to restore homeostasis in injured joints and prevent secondary osteoarthritis.

## Background

Synovium is a membrane that lines the cavity of synovial joints and consists of a lining layer of macrophage-like synoviocytes and fibroblast-like synoviocytes (FLS), as well as a sublining of connective tissue [1]. Synovial cells are thought to contribute to joint homeostasis by secreting various factors such as hyaluronic acid and lubricin important for joint lubrication and function, as well as disposing of the waste products. They respond to injuring factors such as trauma and inflammation (arthritis), with aberrant proliferation of stromal cells resulting in synovial hyperplasia, which can become clinically prominent by causing pain and swelling.

Joint injury is a known risk factor for secondary osteoarthritis [2] and triggers an accompanying synovial hyperplasia whose biological events remain poorly understood. An understanding of the events occurring during synovial hyperplasia in joint pathophysiology will ultimately impact the development of therapeutic interventions for osteoarthritis [3].

We recently provided evidence that mesenchymal stromal/stem cells (MSCs) are key players in synovial hyperplasia secondary to joint surface injury [4]. First isolated from bone marrow [5, 6], MSCs were later established in vitro from several connective tissues as fibroblast-like cells with abilities to form colonies derived from single cells (colony-forming unit fibroblasts [CFU-F]) and to give rise to mature cells of mesenchymal lineages such as osteoblasts and chondrocytes [7]. We previously reported the isolation and characterisation of multipotent MSCs from the adult human synovium [8–11]. More recently, we employed a double-nucleoside analogue labelling method to demonstrate the in vivo identification and location of MSCs in the mouse synovium, and showed that following joint surface injury these cells proliferated to sustain synovial hyperplasia [4]. Whether these proliferative MSCs in hyperplastic synovium are resident synovial cells or move into the tissue from elsewhere is not known.

There is evidence that bone marrow cells can migrate into synovium, raising the possibility that an influx of bone marrow-derived cells may contribute to the pool of proliferating MSCs during injury-induced synovial hyperplasia [12–14]. In an early study by Edwards et al. [12], wild-type mice subjected to beige mouse-derived bone marrow transplants showed the emergence in their synovium of donor-derived cells, which appeared to be macrophage-like synoviocytes on the basis of their morphology as analysed by electron microscopy. However, the detection rate of the characteristic large granules in synovium of beige donor mice themselves was less than one in ten cells, and it was not clear whether the large granules were more prevalent or readily detectable in cells of the monocyte lineage [12]. In more recent studies, researchers have investigated the influx of bone marrow cells into synovium under inflammatory conditions using murine models of rheumatoid-like inflammatory arthritis. Marinova-Mutafchieva et al. [13] identified a population of mesenchymal progenitor cells on the basis of expression of bone morphogenetic protein receptor types 1A and 2 (BMPR1A and BMPR2, respectively), which were not found in normal synovium but accumulated in synovium in the collagen-induced arthritis (CIA) model. BMPR-expressing cells were present in epiphyseal bone marrow of control mice and were suggested to infiltrate the synovium of CIA mice through enlarged bone canals connecting the two tissues [13]. Li and Makarov [14] transplanted green fluorescent protein (GFP)-labelled bone marrow cells into wild-type mice and then induced antigen-induced arthritis. Primary FLS cultures established from arthritic joints and expanded to passage 3 contained over 30 % of GFP^+^ (bone marrow-derived) cells, significantly higher than the approximately 1 % observed in cultures obtained from healthy joints. On the basis of their plastic adherence, the donor-derived cells in the FLS cultures were suggested to be mesenchymal cells, though no phenotypic characterisation was presented [14].

Altogether, these studies suggest that under normal conditions there is minimal, if any, migration of mesenchymal cells from bone marrow to synovium, and that in rheumatoid-like inflammatory arthritis an influx of bone marrow-derived mesenchymal cells may contribute to synovial pannus formation. Whether such an influx of bone marrow cells contributes to the synovial hyperplasia accompanying non-inflammatory joint disorders such as trauma is not known.

In this study, we set out to investigate the contribution of bone marrow MSCs to synovial hyperplasia following joint surface injury. To this end, we established a mouse MSC chimeric model via transplant of genetically labelled bone marrow into lethally irradiated mice and characterised bone marrow-derived cells in the synovium of normal knee joints and after joint surface injury [15]. Our study provides evidence that the majority of the MSCs that proliferate after injury are resident synovial cells, with a contribution to the synovial hyperplasia from increased infiltration of predominantly haematopoietic bone marrow-derived cells.

## Methods

### Animals

The animal experiments were approved by the U.K. Home Office and conducted in accordance with the U.K.’s Animals (Scientific Procedures) Act 1986 and the U.K. Home Office Code of Practice. Wild-type C57BL/6 mice were purchased from Charles River Laboratories (Edinburgh, UK). C57BL/6-Tg14(act-EGFP)OsbY01 mice (referred to hereafter as *Act-eGFP*), which express enhanced GFP ubiquitously [16], were bred in our animal facility (originally obtained from Prof. Okabe, Genome Information Research Centre, Osaka University, Osaka, Japan).

### Bone marrow transplant

Bone marrow transplants were performed as previously described [17]. Mice (7- to 9-week-old females) received full-body irradiation in two doses of 5 Gy given 3 h apart. The donor cells were isolated by flushing bone marrow cells from the femur, tibia, and pelvis with PBS supplemented with 2 % foetal calf serum (FCS). A total of 5 × 10^6^ donor cells per mouse were delivered 24 h after irradiation via tail vein injection. Chimerism was determined in a peripheral blood sample obtained from the tail vein at 3 weeks post-transplant using flow cytometry.

### Joint surface injury

The joint surface injury procedure was performed as previously described [15]. Briefly, 3 to 4 weeks after bone marrow transplant, mice were anaesthetised and subjected to surgery under a dissection microscope. An incision was made to open the skin over the knee joint area, followed by an incision along the medial side of the patellar ligament and through the quadriceps muscle to aid patellar dislocation. The patellar groove was exposed and a full-cartilage-thickness scratch along the length of the groove was made using a 25-gauge needle. The patella was then relocated, and the joint capsule and the skin were sutured. The contralateral knee served as an uninjured internal control. Sham-operated knee joints were subjected to arthrotomy, but no cartilage injury was made.

### Nucleoside analogue administration

Nucleoside analogue administration was performed as previously described [4]. Briefly, 4-week-old mice received iododeoxyuridine (IdU; Sigma-Aldrich, Dorset, UK) in drinking water (1 mg/ml) for 3 weeks, followed by a 5-week wash-out period. Immediately after joint surface injury, mice received an injection of chlorodeoxyuridine (CldU; Sigma-Aldrich) subcutaneously (10 mg/ml) and then received CldU in their drinking water (1 mg/ml) for 7 days.

### Bone marrow cell isolation

Bone marrow cells were isolated as previously described [18]. Briefly, mouse limbs were dissected and muscles were removed using a scalpel. Bones were then cut into small fragments using scissors, washed three times with HBSS+ buffer (Hank’s balanced salt solution [HBSS] supplemented with 2 % FCS, 10 mM HEPES, and 1 % penicillin [10,000 U/ml]/streptomycin [10 mg/ml]) and incubated in pre-warmed DMEM supplemented with 0.2 % collagenase type I (Sigma-Aldrich), 10 mM HEPES and 1 % penicillin (10,000 U/ml)/streptomycin (10 mg/ml) for 1 h in a water bath shaken at 140 rpm. Samples were vortexed every 10 minutes to assist cell dissociation. After collagenase incubation, the supernatant was collected through a 70-μm filter, and bones were further crushed in a mortar with pestle with approximately 50 taps. The crushed bone fragments were washed with HBSS+ buffer several times until clear, and all collected cells were pelleted. After red blood cells were lysed using BD Pharm Lyse buffer (BD Biosciences, Oxford UK), cells were washed and viable cells were counted using trypan blue exclusion.

### CFU-F assay

Bone marrow cells were seeded at 2.5 × 10^4^ cells/cm^2^ and maintained in MesenCult MSC Basal Medium supplemented with MesenCult Stimulatory Supplements (STEMCELL Technologies, Vancouver, BC, Canada) in accordance with the manufacturer’s instructions for 2 weeks, after which the number of CFU-F was scored. Clusters of ≥32 cells were considered colonies. Thirty-two cells were chosen because this represents a population of cells derived from at least five population doublings of a single cell, thereby discounting transit-amplifying cells [19]. GFP fluorescence was detected using an EVOS FL microscope (Life Technologies, Carlsbad, CA, USA).

### Flow cytometry

Mononuclear cells from peripheral blood were stained with CD45-peridinin chlorophyll protein complex (PerCP)-cyanine 5.5 (Cy5.5) antibody (clone 30-F11; eBioscience, San Diego, CA, USA). Freshly isolated bone marrow cells were co-stained with CD45-phycoerythrin (clone 30-F11; BD Biosciences), CD140a-allophycocyanin (clone APA5; BD Biosciences) and stem cell antigen 1 (Sca-1)-PerCP-Cy5.5 (clone D7; eBioscience) antibodies for 30 minutes at 4 °C. Cells were analysed using a BD LSR II flow cytometer (BD Biosciences). Dead cells were excluded from the analysis based on staining with eFluor 780 viability dye (65-0865; eBioscience).

### Histology

Knee joints were fixed in 4 % methanol-free paraformaldehyde (TAAB Laboratories Equipment, Aldermaston, UK) for 24 h at 4 °C and then decalcified in 10 % ethylenediaminetetraacetic acid (EDTA) (VWR International, Lutterworth, UK) in PBS for 2 weeks. The samples were then either embedded in paraffin and cut into 5-μm sections using a standard microtome (Leica RM2125 RT; Leica Biosystems, Milton Keynes, UK) or, to directly visualise GFP under a fluorescence microscope incubated sequentially in 15 % and 30 % sucrose in PBS, embedded in O.C.T. compound (VWR International) over liquid nitrogen and cut into 8-μm sections using a cryostat (Leica CM1850 UV; Leica Biosystems). Haematoxylin and eosin (H&E) staining was performed according to standard protocols.

### Immunofluorescence staining

Immunofluorescence staining of paraffin sections was performed as previously described [4], with some modifications. Antigen retrieval was either (1) enzyme-based by incubation with 1 mg/ml to 3 mg/ml porcine pepsin (Sigma-Aldrich) for 45 minutes at 37 °C or (2) heat-mediated in an EDTA-based solution (pH 9; Vector Laboratories, Burlingame, CA, USA) or citrate buffer solution (pH 6; Vector Laboratories) for 30 minutes in a steamer. Autofluorescence was quenched by incubation in 1 % Sudan Black B (Sigma-Aldrich) solution (in 70 % ethanol) for 20 minutes at room temperature, and non-specific binding was blocked with 1 % bovine serum albumin (Sigma-Aldrich) for 1 h. Sections were then incubated with primary antibodies (listed in the subsection immediately below) overnight at 4 °C in a humidified chamber, followed by incubation with Alexa Fluor 488- or Alexa Fluor 594-conjugated secondary antibodies (Life Technologies) for 1 h at room temperature in the dark. The nuclei were counterstained with 4′,6-diamidino-2-phenylindole (DAPI, Life Technologies).

### Antibodies

The following primary antibodies were used: anti-GFP (chicken polyclonal antibody; Abcam, Cambridge, UK), anti-GFP (rabbit monoclonal antibody; Abcam), anti-bromodeoxyuridine (rat monoclonal antibody, clone ICR1, cross-reacts with CldU but not with IdU; Abcam), anti-IdU (mouse monoclonal antibody, clone 32D8.D9; Abcam), anti-von Willebrand factor (anti-vWF) (rabbit polyclonal antibody; Dako, Ely, UK), anti-platelet-derived growth factor receptor α (anti-Pdgfrα) (rabbit polyclonal antibody; Abcam) and anti-CD16/CD32 (goat monoclonal antibody; R&D Systems, Minneapolis, MN, USA).

### Imaging and cell counting

Images of both cryosections and paraffin-embedded sections were acquired using a Zeiss LSM 710 confocal microscope (Carl Zeiss Microscopy, Jena, Germany) for fluorescence analysis or a Zeiss Imager Axioskop 40 microscope (Carl Zeiss Microscopy) equipped with a ProgRes C14 camera (Jenoptik, Jena, Germany) for H&E-stained sections. Image processing and manual cell counting were performed using ZEN 2010 (Carl Zeiss Microscopy) or ImageJ (National Institutes of Health, Bethesda, MD, USA) software. For each condition, three sections per knee joint from at least three mice were analysed. A minimum of 200 total cells from each mouse knee joint were counted. To avoid the confounding influence of the medial parapatellar arthrotomy in injured knee joints, quantification was carried out in the lateral compartment of the knee.

### Statistics

Data were analysed using IBM SPSS (IBM, Armonk, NY, USA) and Prism (GraphPad Software, La Jolla, CA, USA) software. Statistical significance was determined using either *t* tests for two-group comparisons, or two-way analysis-of-variance and the Bonferroni post-hoc test for multi-group comparisons, with *p* < 0.05 considered significant.

## Results

### Synovial hyperplasia following joint surface injury

**Fig. 1:**
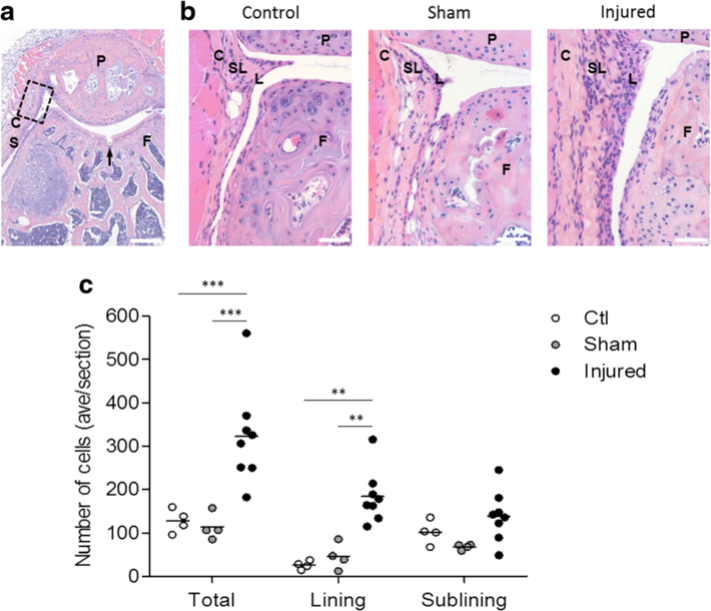
Synovial hyperplasia following joint surface injury. **a** Haematoxylin and eosin (H&E)-stained histological section of an injured 14-week-old mouse knee joint. *Boxed area* indicates the area of synovium analysed in **b** and **c**. *Arrow* indicates joint surface injury. *P* patella, *F* femur. Scale bar = 800 μm. **b** H&E-stained histological sections of uninjured (control), sham-operated and injured knee joint synovium showing hyperplasia 6 days after joint surface injury. *L* lining, *SL* sublining, *C* capsule, *P* patella, *F* femur. Scale bars = 100 μm. **c** Number of cells in the total, lining and sublining of synovium, quantified from images as in **b**, showing a significant increase in cellularity in both the total synovium and the synovial lining of injured but not sham-operated knees, as compared with uninjured controls. Data are expressed as the average number of cells per quantified histological section and shown as individual data points from four (uninjured and sham controls) or eight (injured) mice, with *horizontal lines* indicating mean values. **p < 0.01; ***p < 0.001

We previously showed extensive proliferation of label-retaining cells with an MSC phenotype in the synovium of knee joints challenged with an injury to the articular surface of the femoral groove, while little proliferation was observed in the synovium of sham-operated knees [4]. To confirm that the synovial hyperplasia in this model is specific to the joint surface injury, the synovium near the patellofemoral junction of uninjured, sham and injured knee joints was evaluated by H&E staining (Fig. 1a and b). Of note, the surgical incision to access the joint was made on the medial side of the knee, while the lateral side was analysed to assess the synovial response to injury. Cellularity in injured knee joint synovium increased 2.5-fold compared with uninjured controls (*p* < 0.001) and 2.8-fold compared with sham-operated controls (*p* < 0.001). The increase in cellularity was observed predominantly in the lining (*p* < 0.01). Importantly, the cellularity of synovium from sham-operated knees did not significantly change compared with uninjured control knees (Fig. 1c). Taken together, these data show that synovial hyperplasia in the lateral compartment of the knee occurs in response to the injury of the joint surface and not the arthrotomy.

### Mesenchymal lineage chimerism after bone marrow transplant

To investigate the contribution of bone marrow-derived cells to synovial hyperplasia following joint surface injury, we used the bone marrow transplant technique to label bone marrow-derived cells [17]. Wild-type C57BL/6 mice were lethally irradiated and subjected to transplant of GFP-labelled bone marrow cells derived from transgenic Act-eGFP mice. At 3 weeks after transplant, more than 90 % of CD45^+^ cells in peripheral blood were GFP-labelled (data not shown), thereby confirming successful haematopoietic lineage chimerism.

**Fig. 2:**
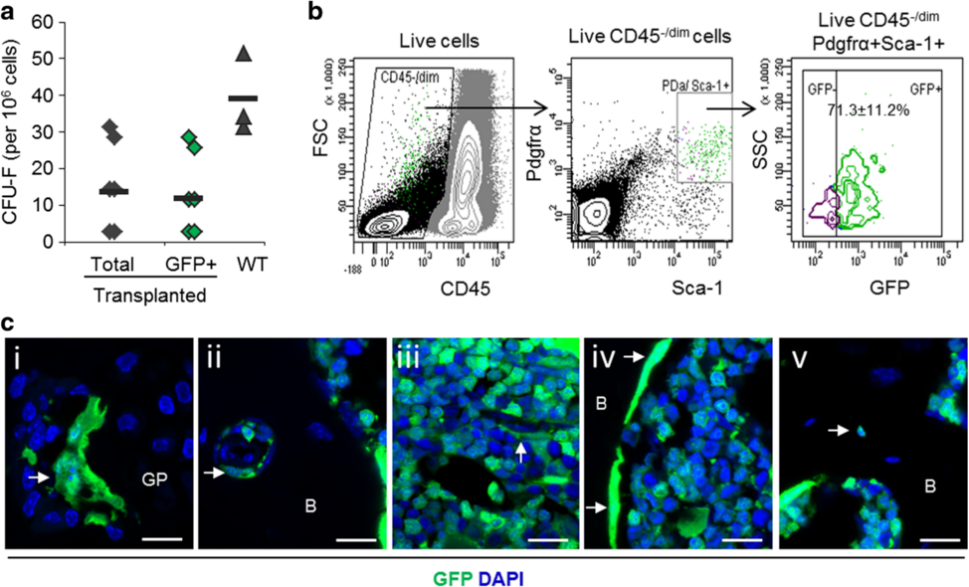
Assessment of bone marrow stromal cell chimerism. C57BL/6 mice received total body irradiation and were subjected to transplant with GFP-labelled bone marrow from C57BL/6-Tg14(act-EGFP)OsbY01 donor mice. Mesenchymal stromal cell lineage chimerism was determined by CFU-F assay, phenotypic analysis of bone marrow cells and histology after 8 weeks. **a** CFU-F assay results showing that the vast majority of CFU-F were GFP^+^ (donor-derived). Data are shown as individual data points from six mice for the transplanted group and three mice for the WT control group. **b** GFP positivity in the CD45^−/dim^Pdgfrα^+^Sca-1^+^ bone marrow MSC population. Shown are representative flow cytometry plots indicating gating strategy. Gating for GFP was based on a WT mouse sample. The percentage of CD45^−/dim^Pdgfrα^+^Sca-1^+^ cells positive for GFP (donor-derived) is shown as mean ± SD (*n* = 7). **c** Confocal microscopic images showing a GFP^+^ osteoclast (arrow) in the growth plate (GP) region (*i*), GFP^+^ perivascular cells (arrows) in bone (B) (*ii*) and bone marrow (*iii*), GFP^+^ bone lining cells (arrows) at the endosteal surface (*iv*) and a GFP^+^ osteocyte (arrow) embedded in bone matrix (*v*). Scale bars = 25 μm. *CFU-F* colony-forming unit fibroblasts, *FSC* forward scatter, *Pdgfrα* platelet-derived growth factor receptor α, *Sca-1* stem cell antigen 1, *SSC* side scatter, *WT* wild-type, *GFP* green fluorescent protein, *GP* growth plate, *B* bone, *MSC* mesenchymal stromal/stem cell

To determine the degree of MSC chimerism in the bone marrow stromal compartment, we employed a standard CFU-F assay using freshly isolated bone marrow cells (Fig. 2a). The numbers of CFU-F ranged from 3 to 31 per 10^6^ bone marrow cells (average 16 CFU-F/10^6^ cells, *n* = 6). When analysed for GFP expression, approximately 90 % of the colonies were GFP^+^. Bone marrow derived from control mice that did not undergo transplants contained 39 ± 11 CFU-F per 10^6^ cells (*n* = 3) (Fig. 2a).

To further determine whether MSCs in bone marrow were successfully labelled, freshly isolated (uncultured) MSCs were identified on the basis of their CD45^−/dim^Pdgfrα^+^Sca-1^+^ phenotype, as previously reported [18]. The percentage of GFP^+^ cells within the Pdgfrα^+^Sca-1^+^ MSC fraction, which constituted 0.14 ± 0.07 % of isolated CD45^−/dim^ cells, was 71.3 ± 11.2 % (*n* = 7) (Fig. 2b). Bone marrow chimerism was confirmed by histological analysis, which showed the presence of GFP-labelled cells not only within the haematopoietic compartment (including large, multinucleated osteoclasts; Fig. 2c-i) but also in the bone marrow stroma, including perivascular cells (Fig. 2c-ii, iii), bone lining cells covering the endosteal surfaces (Fig. 2c-iv) and osteocytes embedded within the bone matrix (Fig. 2c-v). Taken together, the CFU-F and phenotypic studies of freshly isolated bone marrow cells and the histological analyses of cryosections demonstrated efficient mesenchymal lineage chimerism in the bone marrow of transplanted mice.

### Bone marrow-derived cells in the synovium of mice that received transplants

**Fig. 3:**
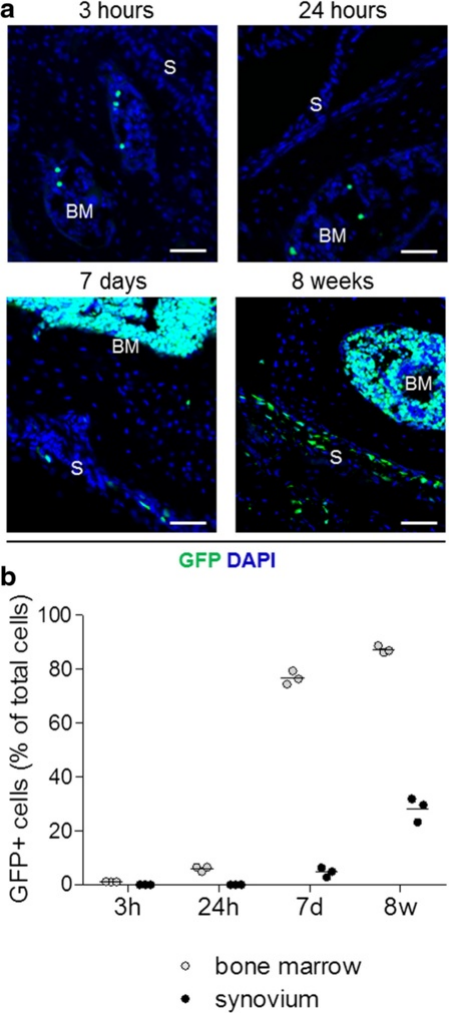
Detection of green fluorescent protein-positive (GFP^+^) cells in the synovium of bone marrow chimeric mice. **a** Representative confocal microscopic images of knee joint cryosections showing GFP^+^ (*green*) cells in the bone marrow (BM) and synovium (S) with 4′,6-diamidino-2-phenylindole (DAPI) (*blue*) counterstaining at multiple time points following bone marrow transplant as indicated. Scale bars = 50 μm. **b** Quantification of donor-derived GFP^+^ cells in bone marrow and synovium as percentages of total nucleated cells at the indicated time points post-transplant. Data are shown as individual data points from three mice. *Horizontal lines* indicate mean values. *p* < 0.0001 by two-way analysis of variance of bone marrow versus synovium

We next monitored over time the numbers of donor-derived GFP^+^ cells in bone marrow and synovium. GFP^+^ cells were detected in the bone marrow as early as 3 h post-transplant and increased to 76.8 ± 2.5 % of cells at 7 days (Fig. 3), showing the rapid homing to and reconstitution of the bone marrow by systemically injected donor cells, as expected. In contrast, GFP^+^ cells were not detected in the synovium at the early 3- and 24-h time points. They were first detected in small numbers at 7 days (4.7 ± 1.8 % of total cells) and increased to 28.3 ± 4.4 % of total cells at 8 weeks (Fig. 3). These data suggest a gradual, continuous migration of bone marrow-derived cells into the synovium, which occurs after the initial homing of injected donor cells to bone marrow.

### Contribution of bone marrow-derived cells to synovial hyperplasia following joint surface injury

**Fig. 4:**
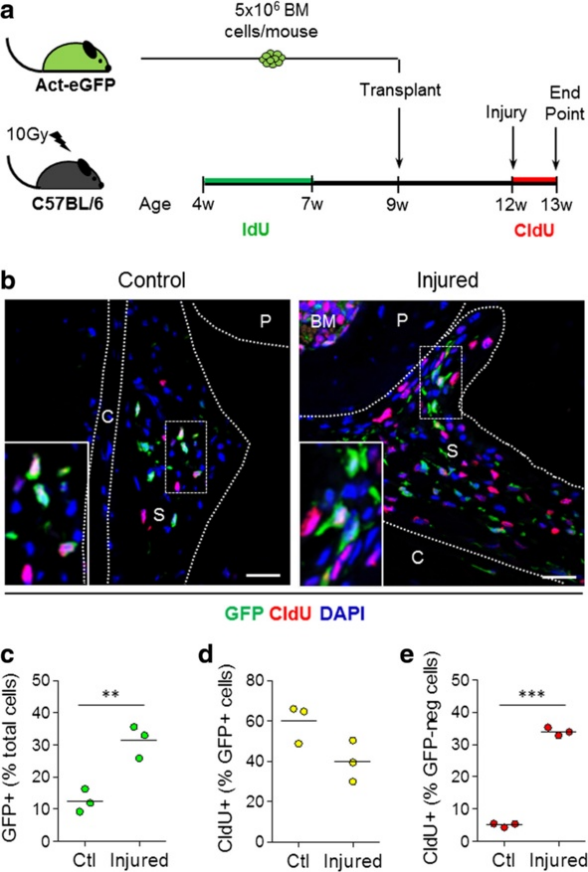
Detection of GFP^+^ cells in synovium of bone marrow chimeric mice following joint surface injury. **a** Schematic diagram showing experimental design for the data shown in this figure and Fig. 5. **b** Representative images of immunofluorescent staining for GFP (*green*) and the proliferation marker CldU (*red*) in control and injured knee joint synovium. Nuclei are counterstained with 4′,6-diamidino-2-phenylindole (DAPI; *blue*). Scale bars = 25 μm. The *insets* show magnified views of the areas within *dotted rectangles. BM* bone marrow, *S* synovium, *C* capsule, *P* patella, *F* femur. **c**–**e** Quantification of GFP and CldU single- and double-positive cell populations in the synovium. Data are shown as individual data points from three mice, with *horizontal lines* indicating mean values. **p < 0.01; ***p < 0.001. *Act-eGFP* C57BL/6-Tg14(act-EGFP)OsbY01 mice, *CldU* chlorodeoxyuridine, *GFP* green fluorescent protein, *IdU* iododeoxyuridine

To assess the contribution of bone marrow-derived cells to synovial hyperplasia after joint trauma, chimeric mice carrying GFP-labelled bone marrow were challenged with joint surface injury. Double-nucleoside labelling [4] was used to identify host-derived MSCs by giving mice the nucleoside analogue IdU for 3 weeks prior to bone marrow transplant. Immediately after injury, mice received a second nucleoside analogue, CldU, for 7 days to label proliferating cells until tissues were collected for analysis (Fig. 4a). Immunofluorescent staining of paraffin-embedded knee joint sections was used to detect labelled cells.

We initially focussed on the GFP-labelled, bone marrow-derived donor cells (Fig. 4b). Quantitative analysis revealed a significant increase in the percentage of GFP^+^ cells in injured (31.5 ± 5.1 %, *n* = 3) compared with contralateral control knee joint synovium (12.5 ± 3.6 %, *n* = 3; *p* < 0.01) (Fig. 4c), demonstrating an expansion of the bone marrow-derived cell population in synovium after injury. However, the percentage of GFP^+^ cells that were positive for the proliferation marker CldU did not change significantly following injury (Fig. 4d), suggesting that the increase in GFP^+^ cells in synovium was due mainly to increased infiltration of donor-derived cells rather than a proliferative response to the injury. In contrast, the percentage of GFP^−^ cells that were CldU^+^ significantly increased (*p* < 0.001) (Fig. 4e).

**Fig. 5:**
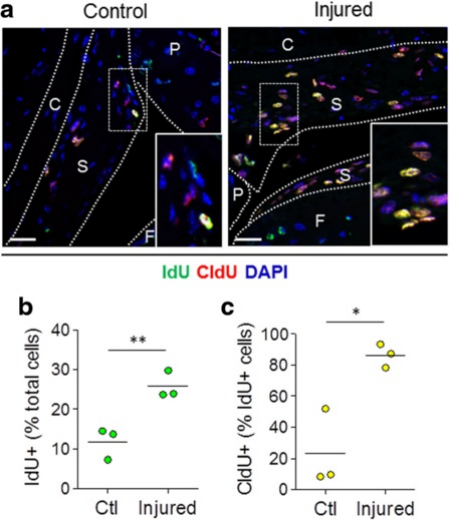
Proliferation of host slow-cycling cells in synovium following joint surface injury. The schematic diagram of the experimental design is shown in Fig. 4a. **a** Representative images of immunofluorescent staining for iododeoxyuridine (IdU, *green*) and chlorodeoxyuridine (CldU, red) in control and injured knee joint synovium. Nuclei are counterstained with 4′,6-diamidino-2-phenylindole (DAPI, *blue*). Scale bars = 25 μm. The *insets* show magnified views of the areas within *dotted rectangles. S* synovium, *C* capsule, *P* patella, *F* femur. **b**, **c** Quantification of nucleoside analogue-labelled cells in the synovium. Data are shown as individual data points from three mice, with *horizontal lines* indicating mean values. *p < 0.05; **p < 0.01

To ascertain that the cells in synovium that proliferated after injury were host-derived, label-retaining cells, we performed double-immunofluorescence staining for IdU and CldU (Fig. 5a). Quantitative analysis showed that the percentage of total IdU^+^ cells increased significantly from 11.8 ± 4.0 % in the synovium of uninjured control joints to 25.8 ± 3.5 % in injured joints (*p* < 0.01, *n* = 3) (Fig. 5b). Moreover, the percentage of proliferative CldU^+^ cells within the IdU^+^ population strongly increased (*p* < 0.05, *n* = 3) (Fig. 5c). This confirmed that the label-retaining cells from the host retained their ability to proliferate and were major contributors to the synovial hyperplasia in the bone marrow transplant model.

### Phenotypic characterisation of bone marrow-derived cells in the synovium

**Fig. 6:**
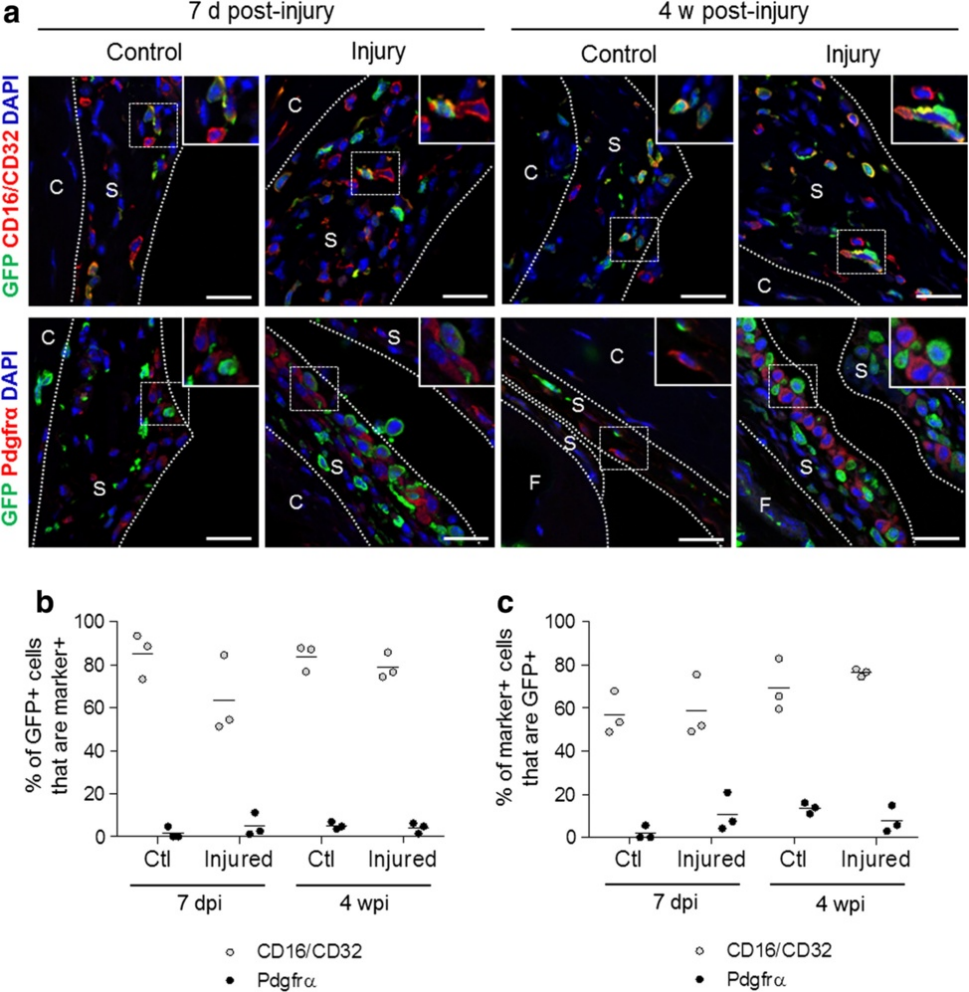
Phenotypic characterization of GFP^+^ cells in synovium. **a** Paraffin-embedded sections from control and injured knee joints at 7 days post-injury (7dpi) and 4 weeks post-injury (4wpi) were subjected to immunofluorescence staining for GFP (*green*) in conjunction with CD16/CD32 (*red*) or Pdgfrα (*red*) and counterstained with 4′,6-diamidino-2-phenylindole (DAPI, *blue*). Scale bars = 25 μm. The *insets* show magnified views of the areas within *dotted boxes. S* synovium, *C* capsule, *P* patella, *F* femur. **b**, **c** GFP^+^CD16/CD32^+^ cells and GFP^+^Pdgfrα^+^ cells in the synovium shown as percentages of (**b**) the total GFP^+^ cell population and (**c**) the total marker-positive cell population. Data are shown as individual data points from three mice, with *horizontal lines* indicating mean values. *GFP* green fluorescent protein, *Pdgfrα* platelet-derived growth factor receptor α

We investigated the phenotype of bone marrow-derived GFP^+^ cells in the synovium by immunofluorescence staining for GFP in conjunction with cell lineage markers (Fig. 6a). The majority of GFP^+^ cells in the control synovium of uninjured joints expressed the haematopoietic marker CD16/CD32, known to be expressed by macrophages in the synovial lining [20], while a very small number of GFP^+^ cells were positive for the pan-fibroblast/MSC marker Pdgfrα [18, 21] (Fig. 6b). After injury, the majority of GFP^+^ cells in synovium remained positive for CD16/CD32, with a minor contribution from Pdgfrα^+^ MSCs/fibroblasts, both at 7 days post-injury (7dpi), when synovial hyperplasia was florid, and at 4 weeks post-injury (4wpi), when hyperplasia had decreased (Fig. 6b). Conversely, a substantial proportion of the CD16/32^+^ haematopoietic cells in synovium were donor-derived in both control and injured knee joints, while the proportion of GFP-labelled cells within the Pdgfrα^+^ MSC/fibroblast population was low and remained low after injury, both at 7dpi and 4wpi (Fig. 6c). No co-localisation of GFP and the endothelial cell marker vWF was detected in synovium (data not shown). Taken together, these findings indicate that the GFP^+^ bone marrow-derived cells in the synovium are mostly of haematopoietic lineage.

## Discussion

Synovial hyperplasia is a frequent clinical finding in patients with joint disorders. Its pathogenesis is likely to be distinct, depending on the underlying clinical condition. While it is driven in large part, if not entirely, by inflammation/autoimmunity in rheumatoid arthritis, its pathogenesis in patients with joint trauma or osteoarthritis is not so well defined [22]. We recently identified the MSC as a key cell player in synovial hyperplasia secondary to joint surface injury in mice by demonstrating that label-retaining cells with an MSC-like phenotype undergo a burst of proliferation, which results in an expansion of the MSC pool associated with a thickening of the synovium [4]. Human synovium is also known to contain MSCs [8–11], raising the possibility that a similar cell population may underpin the synovial hyperplasia in patients after joint trauma. However, bone marrow is also well known to harbour MSCs [7] and is connected to the synovium and joint space via bone canals. It is therefore conceivable that an influx of cells from bone marrow into synovium could contribute to the pathogenesis of synovial hyperplasia, as suggested by previous studies in rodent models of inflammatory arthritis [13, 14].

In the present study, we show that hyperplasia of the synovium in the lateral compartment of the knee joint following surgically induced injury that involved medial arthrotomy to access the joint specifically occurred in response to the joint surface damage, as no hyperplasia was observed in the lateral synovium of sham-operated knee joints. The increased cellularity of the synovium after joint surface injury was most notable in the synovial lining.

To investigate the contribution of bone marrow cell populations to synovial hyperplasia secondary to joint surface injury, we carried out transplant of genetically labelled bone marrow into lethally irradiated wild-type mice. Following the bone marrow transplant protocol outlined by Koide et al. [17] and Morikawa et al. [18], we achieved comparable bone marrow stromal cell compartment reconstitution with genetically labelled donor cells as assessed by CFU-F assay, analysis of the CD45^−^Pdgfrα^+^Sca-1^+^ MSC population and histological cell assessment. The CFU-F assay indicated around 90 % efficiency of stromal cell chimerism in bone marrow, while the flow cytometry analysis of the CD45^−^Pdgfrα^+^Sca-1^+^ MSC population revealed approximately 70 % of the contribution to be from the donor. This difference could be due to different sensitivities of the assays used to detect MSCs or to host cells losing their proliferative capacity in vitro as a consequence of the total body irradiation prior to bone marrow transplant [18]. The latter, however, did not appear to be true for the stromal cells in synovium, which displayed a high degree of proliferation after injury similar to what we reported previously in non-irradiated mice [4]. The efficiency of the stromal cell reconstitution following bone marrow transplant was further corroborated by the detection of donor-derived cells along the mesenchymal lineage, including bone lining cells and osteocytes, and cells in a perivascular location, a known niche for MSCs in bone marrow [23–25].

Trafficking of bone marrow-derived MSCs into organs and tissues has been reported to occur in intestine and muscle [26], and also in skin [27]. Studies of the contribution of bone marrow-derived MSCs to synovium are limited, and mostly in the context of inflammatory/autoimmune arthritis. Marinova-Mutafchieva et al. [13] reported accumulation in the synovium of rheumatoid-like CIA mice of BMPR-expressing mesenchymal progenitor cells, not detected in normal synovium, an accumulation that was thought to occur through infiltration from bone marrow via enlarged bone canals. Likewise, Li and Makarov [14] reported an increase in bone marrow-derived MSC contribution to the synovium during antigen-induced inflammatory arthritis using a GFP-labelled bone marrow transplant model in wild-type mice. We employed a similar chimeric model to focus our analysis on the synovial hyperplasia secondary to joint surface injury, and to this end we elected to use a clinically relevant knee joint trauma model that is not driven by overt inflammatory or autoimmune events [15]. Our findings showing an influx of bone marrow-derived cells into a trauma-induced hyperplastic synovium are novel in the context of joint trauma and, due to different aetiopathogenesis, cannot be compared with those obtained using inflammatory/autoimmune models of synovial hyperplasia (synovitis).

The increase in bone marrow-derived cells in synovium following joint surface injury was likely due to enhanced infiltration of cells from the bone marrow and/or circulation rather than increased proliferation in situ, since the proportion of donor-derived cells in synovium that proliferated did not increase after injury. We monitored the transplanted cells over time to ascertain that the systemically injected donor cells did not immediately home to the synovium, but rather gradually infiltrated the synovial tissue over the course of several weeks.

Most of the GFP^+^ cells in synovium were of haematopoietic lineage (CD16/32^+^), while few expressed the pan-fibroblast/MSC marker Pdgfrα [18, 21]. Interestingly, while there was an overall increase in bone marrow-derived GFP^+^ cells in synovium following injury, the proportions of haematopoietic and stromal cells remained similar, indicating there was no clear preferential or selective expansion of one cell population over other cell populations. This would support homing as a stochastic event, which could be explained by increased vascularisation and blood supply. Nonetheless, specific molecular mechanisms underpinning the inflow of mesenchymal cells from bone marrow into synovium could still be at play. Recent work has demonstrated that placental growth factor, whose levels are increased in rheumatoid arthritis joints, could recruit bone marrow MSCs to the synovium [28].

## Conclusions

Taken together, our findings show a dual contribution to the synovial hyperplasia following joint surface injury from infiltration of bone marrow-derived cells, which include haematopoietic cells and a minority of Pdgfrα^+^ MSCs/fibroblasts, and proliferation of MSCs that are likely tissue-resident. MSCs resident in synovium and bone marrow-derived MSCs infiltrating the synovium could be functionally distinct MSC subpopulations. Lineage tracing studies will provide further insights into the existence of distinct MSC subpopulations, their specialised functions and their possible plasticity in the context of joint health and disease.

## Abbreviations

Act-eGFP, C57BL/6-Tg14(act-EGFP)OsbY01 mice; BM, bone marrow; BMPR, bone morphogenetic protein receptor; CFU-F, colony-forming unit fibroblasts; CIA, collagen-induced arthritis; CldU, chlorodeoxyuridine; Cy5.5, cyanine 5.5; DAPI, 4′,6-diamidino-2-phenylindole; dpi, days post-injury; EDTA, ethylenediaminetetraacetic acid; FCS, foetal calf serum; FLS, fibroblast-like synoviocytes; FSC, forward scatter; GFP, green fluorescent protein; HBSS, Hank’s balanced salt solution; IdU, iododeoxyuridine; H&E, haematoxylin and eosin; MSC, mesenchymal stromal/stem cell; Pdgfrα, platelet-derived growth factor receptor α; PerCP, peridinin chlorophyll protein complex; Sca-1, stem cell antigen 1; SSC, side scatter; vWF, von Willebrand factor; wpi, weeks post-injury; WT, wild type
